# A Social Recommendation Model Based on Basic Spatial Mapping and Bilateral Generative Adversarial Networks

**DOI:** 10.3390/e25101388

**Published:** 2023-09-28

**Authors:** Suqi Zhang, Ningjing Zhang, Wenfeng Wang, Qiqi Liu, Jianxin Li

**Affiliations:** 1 School of Information Engineering, Tianjin University of Commerce, Tianjin 300134, China; 2 School of Science, Tianjin University of Commerce, Tianjin 300134, China; zhangningjing@stu.tjcu.edu.cn (N.Z.); wangwenfeng@stu.tjcu.edu.cn (W.W.); 3 School of Artificial Intelligence and Data Science, Hebei University of Technology, Tianjin 300401, China; qiqi.liu@hebut.edu.cn; 4 School of IT, Deakin University, Burwood, VIC 3125, Australia; jianxin.li@deakin.edu.au

**Keywords:** recommendation algorithm, social recommendation, generative adversarial network, nonlinear mapping

## Abstract

Social recommender systems are expected to improve recommendation quality by incorporating social information when there is little user–item interaction data. Therefore, how to effectively fuse interaction information and social information becomes a hot research topic in social recommendation, and how to mine and exploit the heterogeneous information in the interaction and social space becomes the key to improving recommendation performance. In this paper, we propose a social recommendation model based on basic spatial mapping and bilateral generative adversarial networks (MBSGAN). First, we propose to map the base space to the interaction and social space, respectively, in order to overcome the issue of heterogeneous information fusion in two spaces. Then, we construct bilateral generative adversarial networks in both interaction space and social space. Specifically, two generators are used to select candidate samples that are most similar to user feature vectors, and two discriminators are adopted to distinguish candidate samples from high-quality positive and negative examples obtained from popularity sampling, so as to learn complex information in the two spaces. Finally, the effectiveness of the proposed MBSGAN model is verified by comparing it with both eight social recommendation models and six models based on generative adversarial networks on four public datasets, Douban, FilmTrust, Ciao, and Epinions.

## 1. Introduction 

With the development and popularity of the internet, people are facing an increasingly serious problem of information overload [1]. As an important information filtering technology, recommendation algorithms can provide users with personalized information that meets their interests and needs, saving their time and improving the efficiency of information utilization. Recommendation algorithms have been used widely in many fields [2,3], for example, e-commerce platforms and music and video streaming services. The emergence of social platforms has sparked some analysis regarding social networks [4]. At the same time, the rise of social networking platforms provides a large amount of user-related data for social recommendation, which can effectively improve recommendation quality and user satisfaction by using social relationships and extracting potential user interest features from them. Therefore, social recommendation technology has become an important research direction and research hotspot in the field of recommendation systems [5,6]. 

Currently, social recommendation models are mainly based on the assumption of homogeneity, users with social relationships have similar interests [7]. However, this assumption is not realistic. In reality, the social behavior of users in the social space and the interaction behavior of users in the interaction space are both diverse and contingent. Therefore, it is believed that the heterogeneous information in the two spaces: the social space and the interaction space, shall not be directly fused [8]. 

Among them, the fusion of heterogeneous information refers to the process of combining and harmonizing data from different sources or formats, such as text, images, videos, and user profiles. For example, in a recommender system, integrating information from various sources like product descriptions, user reviews, and social media data to provide personalized recommendations. For example, a user follows and comments on content related to high-calorie food in the social space, while he or she often searches for and buys sports-related goods in the interaction space. Although these two behaviors may not seem to be directly related, the common feature behind them is the user’s pursuit of healthy living. If the information in the interaction space and social space is directly fused, it may recommend high-calorie food to users and ignore their pursuit of healthy life; thus affecting their user experience. Therefore, directly utilizing users’ social behavior to recommend products can introduce a lot of noise, and how to effectively fuse heterogeneous information has become a fundamental problem in the field of social recommendation. At the same time, how to further capture the common features hidden behind heterogeneous information on the basis of effective fusion of heterogeneous information is a problem that needs to be solved. 

Apart from the fusion of heterogeneous information, social recommendation models also focus on how to better mine the data information in the social and interaction spaces to improve recommendation quality. There are a lot of traditional data information mining strategies such as classification, clustering, generative adversarial network (GAN), and regression. Among these, generative adversarial networks [9] are a powerful deep learning model that can generate data with high similarity and have been used widely in areas such as deep learning [10,11,12]. Among them, the mining of data information involves extracting valuable insights and patterns from a large volume of data. It includes techniques such as data preprocessing, feature extraction, and data analysis. For instance, in the field of customer relationship management, mining customer data to identify patterns of customer behavior and preferences for targeted marketing campaigns. In recent years, more and more researchers have started to explore how GAN can be applied to social recommendation to improve recommendation accuracy. The challenge of generative adversarial networks is the design of adversarial ideas, constructing more effective generators and discriminators, so as to use the generative power of generative adversarial networks. In the scope of social recommendations, GAN can be used to generate candidate items [13] or candidate friends [14], in order to facilitate more accurate recommendations. However, most GAN-based approaches only consider either the social space or the interaction space, failing to capture the bilateral information at the same time. 

The organizational structure is as follows: Section 2 introduces the relevant work; Section 3 introduces the specific implementation process and details of the MBSGAN model; in Section 4, the effectiveness of the model was verified through two sets of comparative experiments; finally, the conclusions, limitations, and potential research directions of this study were summarized.

## 2. Related Work

In this section, two lines of related work are presented, namely, the social relationship-based recommendation model and the generative adversarial network-based recommendation model. 

The user’s social relationship information, as an important factor influencing the user’s decision making, has been widely incorporated into social relationship-based recommendation models to improve the accuracy and performance of recommendation models. SBPR [15] transforms social relations into a kind of weight, which is used to strengthen interaction between users, so as to combine social information and interactive information. Sorec [16] is based on probabilistic matrix decomposition, which decomposes the user–item interaction matrix into two low-dimensional matrices and the authors improved the accuracy and performance of the recommendation model by introducing a social network factor matrix between these two matrices to effectively fuse the social and interaction information. DSCF [17] is based on collaborative filtering, in which an attention layer is adopted to fuse interaction and social information. DiffNet++ [18], as a neural network based approach, aggregates higher-order neighbors in the social network and interaction network to obtain user expressions separately and uses a graph attention mechanism to fuse the two user expressions. All of the social recommendation models mentioned above make recommendations by sharing a unified user expression, which achieves the fusion of the two types of information. The advantage of these models is that sharing user expressions can fill in missing data and improve recommendation effectiveness by integrating information from multiple spaces, especially in situations with sparse data. However, these studies overlook the fact that users typically interact with different goals in the interaction space and social space, and the underlying motivations and influencing factors are different, leading to heterogeneity in interaction and social behavior [19]. To solve the above heterogeneity problem, in some social recommendation models, researchers attempt to learn user feature vectors in the interaction space and social space separately, and use these learned user feature vectors to make recommendations. DASO [20] is based on generative adversarial networks, which fuse interaction information and social information by mapping them to each other’s space. DcRec [21] is a graph neural network-based social recommendation model that separates user information in the social space and item space by contrast learning, and then the user feature vectors in the two spaces are fused for recommendation tasks using an attention-based fusion mechanism. Although the above methods solve the problem of heterogeneity by learning users separately, these two models do not take into account that the interaction and social behavior of users are influenced by their own values and personality characteristics, and the two behaviors also share common characteristics, which cannot completely erase the similarity between them [22]. Continuing with the example in the introduction, considering only the user’s interest in high-calorie food content and their social relationship with fitness influencers separately, without considering the underlying features that connect them, can still lead to incorrect judgments, assuming that users both enjoy eating high-calorie food and following fitness influencers. Therefore, they did not fully utilize the common features behind user interaction and social behavior [23].

Generative adversarial networks have been widely used to learn the distribution of user–item interaction data. Liu et al. [24] proposed solution generates reasonable user–item pairs by the relevance score function and the discriminator discriminates between real user–item pairs and the generator-generated user–item pairs. In CFGAN [25], the generator generates reasonable user purchase vectors, and the discriminator discriminates between the real user purchase vectors and the generator-generated user purchase vectors. GCGAN [26] uses convolutional neural networks to generate user purchase vectors based on CFGAN. RSGAN [13] is a social recommendation model based on generative adversarial networks, in which the generator samples the items that friends of the user frequently interact with, the user’s preferred items, and the discriminator is responsible for distinguishing the items sampled by the generator from the real interaction items, so that the items generated by the generator become closer to the user’s preferences through adversarial training. ESRF [14] is also a social recommendation model based on GAN, in which the generator samples a fixed number of friends, and the discriminator is responsible for distinguishing between the ratings of the items sampled by the generator and the user’s own preferences, and the ratings of the items by the average opinions of the sampled friends. By doing this, the friends generated by the generator become more and more reliable through adversarial training, and the recommendations are assisted by the opinions of the friends. GANRec [27] proposes a negative sampling model based on the generative adversarial network, which improves the accuracy of the recommendation system by using GAN to generate negative samples. However, the above recommendation model only considers the use of the bilateral generative adversarial networks in the interaction space. Therefore, in this paper, we build a bilateral generative adversarial network, and use the generative adversarial network in each space to learn user feature vectors in the social space and interaction space at the same time, so as to improve the accuracy of recommendation algorithms. 

## 3. MBSGAN Model

Users’ values and personality traits developed over time directly influence their interaction and social behavior. The way of fully extracting common features between these two spaces will have a great influence on the social recommendation. Therefore, this paper introduces a base feature space to fuse interaction and social information, which contains common user characteristics behind user interaction and social behavior, such as user values, personality, family background, and education. In addition, we constructed a bilateral generative adversarial network in both spaces in order to deeply explore and learn the complex data information in both spaces. While solving the problem of heterogeneity effectively, this better captures the common features behind the two spaces and utilizes bilateral generative adversarial networks to learn information from both spaces simultaneously. 

### 3.1. Overview of the Model Framework

In this paper, we propose a social recommendation model based on basic spatial mapping and bilateral generative adversarial networks (MBSGAN), called MBSGAN, based on spatial mapping and bilateral generative adversarial networks to utilize the underlying feature space to capture the common features behind user interaction and social behavior. Among them, adversarial learning in the interaction space obtains candidate recommended items by learning the interaction information between users and items, while in the social space, candidate friends are obtained by learning the social information between users and their friends. Both modules are adversarial models, but they are based in different data spaces and have different goals. These two adversarial networks are the core content of bilateral adversarial training in this paper. In MBSGAN, the fusion of interaction and social information through spatial mapping and bilateral generative adversarial networks can help deeply explore the interaction information in their respective spaces, so as to improve the accuracy of recommendations.

**Figure 1:**
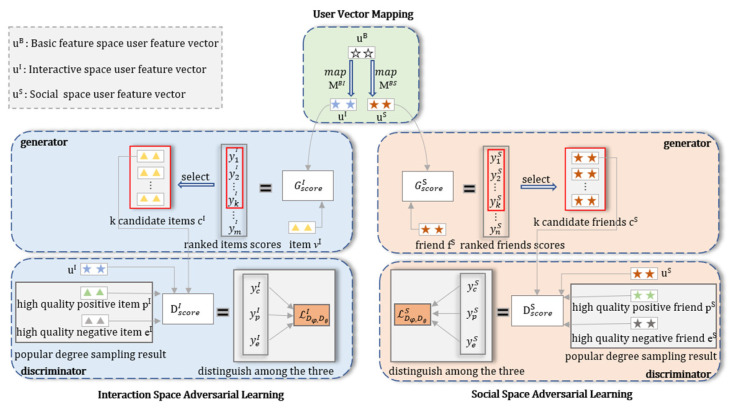
An overview of the proposed MBSGAN framework. *u*^B^, *u*^I^, *u*^S^ represent vector representations of users in the basic feature space, interaction space, and social space, respectively. $v^{I},f^{S},$ respectively, represent the item expression and user friend expression. $c^{I},c^{S}$ represent the candidate items and friends selected by the generator; $p^{I},e^{I},p^{S},e^{S}$ represent high-quality positive and negative examples selected from interactive and social data (please refer to Section 3.3.2 and Section 3.4.2 for detailed interpretation).

The model framework is shown in Figure 1, and the model consists of three modules: a “User Vector Mapping” module, an “Interaction Space Adversarial Learning” module and a “Social Space Adversarial Learning” module.

The “User Vector Mapping” module contains the user’s basic feature vector *u^B^* and two mapping functions *M_B_*_−*I*_ and *M_B_*_−*S*_. First, the user’s base feature vector *u^B^* is mapped through the mapping function *M_B_*_−*I*_ to the interaction space, to obtain the user vector in the interaction space *u*^*I*^. At the same time, the user base feature vector *u^B^* is mapped to the social space by the mapping function *M_B_*_−*S*_ to the social space, to obtain the user expression in the social space *u*^*S*^. Finally, the *u*^*I*^ and *u*^*S*^ are input to the “Interaction Space Adversarial Learning” module and the “Social Space Adversarial Learning” module, respectively, for adversarial training. 

The “Interaction Space Adversarial Learning” module consists of a generator and a discriminator. First, the user feature vector of the interaction space *u*^*I*^ and item vectors *v*^*I*^ are both input into the score function *G*^*I*^_*score*_ (the definition of *G*^*I*^_*score*_ will be given in Equation (4) of Section 3.3). The top k items with the highest scores are selected as candidate items. Then, the user feature vector *u*^*I*^ and the high quality positive items *p*^*I*^, high quality negative items *e*^*I*^ sampled by popularity and the candidate items *c*^*I*^ generated by the generator are input together into the score function $D_{score}^{I}$ (the definition of $D_{score}^{I}$ will be given in Equation (6) of Section 3.3), and then we obtain the correlation scores of users with high-quality positive and negative items *y*_*p*_^*I*^, *y*_*e*_^*I*^ and the correlation scores between the user and the candidate items *y*_*c*_^*I*^. Finally, the loss function $L_{D_{\varphi }}^{I}$ (the definition of $L_{D_{\varphi }}^{I}$ will be given in Equation (8) of Section 3.3) is used to make *y*_*c*_^*I*^ both away from *y*_*p*_^*I*^ and away from *y*_*e*_^*I*^ as far as possible, thus distinguishing the candidate items. 

The “Social Space Adversarial Learning” module also includes a generator and a discriminator. First, the user feature vector of the social space *u*^*S*^ and the friend vector *f*^*S*^ are both input into the score function $G_{score}^{S}$ (the definition of $G_{score}^{S}$ will be given in Equation (11) of Section 3.4), the relevance scores of the user and all friends are obtained, and the top k friends with the highest scores are selected as candidate friends. Then, the user feature vector *u*^*S*^ and the high-quality positive friends *p*^*S*^, high-quality negative friends *e*^*S*^ are sampled by popularity and the candidate friends *c*^*S*^ generated by the generator are input together into the score function $G_{score}^{S}$ to obtain the correlation score between the user and the high-quality positive and negative friends *y*_*p*_^*S*^, *y*_*e*_^*S*^, and the correlation score between the user and the candidate friends *y*_*c*_^*S*^. Finally, the loss function $L_{D_{\varphi }}^{S}$ (the definition of $L_{D_{\varphi }}^{S}$ will be given in Equation (14) of Section 3.4) is used as far as possible to make *y*_*c*_^*S*^ both away from *y*_*p*_^*S*^ and away from *y*_*e*_^*S*^, thus distinguishing the candidate friends. 

After the above bilateral adversarial training process, the candidate items obtained from the interaction space generator are recommended to the user as the items to be recommended. In the following, we will introduce the “User Vector Mapping” module in Section 3.2, the “Interaction Space Adversarial Learning” module and the “Social Space Adversarial Learning” module in Section 3.3 and Section 3.4. Finally, in Section 3.5, we describe the entire adversarial training process of the model. 

### 3.2. “User Vector Mapping” Module

The base feature space is a space that is deeper and more in line with the essence of things than the interaction space and social space. The decisions made by users in any scenario are influenced by their own values, which reflect a user’s orientation and thinking or viewing anything and distinguishing right from wrong, and these values have a certain degree of stability and persistence. Unlike the characteristic factors in social and shopping scenarios, values will not undergo significant changes in a short period of time. Using the base feature space to reflect users’ basic values, and the feature factors of the base feature space can include users’ pursuit of a better life, freedom, and equality, etc. The social and interactive behaviors of users in both social and shopping scenarios are influenced by their own values. Therefore, we believe that the base feature space can be transformed into the interaction space and social space through mapping functions.

We transfer user information from the base feature space (*B*: the basic space) to the interaction space (*I*: the interaction space) and the social space (*S*: the social space) by a nonlinear mapping operation. Specifically, the user’s representation in the base feature space *u*_*i*_*^B^* is mapped to the interaction space and the social space by a mapping function, and the user’s expression in the interaction space *u*_*i*_^*I*^ and the user’s expression in the social space *u*_*i*_^*S*^ are obtained. As shown in Equation (1), the nonlinear mapping function from the base feature space to the interaction space is defined as follows:

(1) $$u_{i}^{I}=M_{p-I}\left(u_{i}^{B}\right)=W_{L}^{I}\cdot \left(\cdots \alpha \left(W_{2}^{I}\cdot \alpha \left(W_{1}^{I}\cdot u_{i}^{B}+b_{1}^{I}\right)+b_{2}^{I}\right)\cdots \right)+b_{L}^{I}$$

In the above equation, the *W*_*S*_^*I*^ and *b*_*S*_^*I*^ are the weights and biases of the *L* layer neural network (the number of layers in this article is set to 2), respectively, and *α* is the nonlinear activation function. Similarly, the nonlinear function from the underlying feature space to the social space is shown in Equation (2):

(2) $$u_{i}^{S}=M_{p-S}\left(u_{i}^{B}\right)=W_{L}^{S}\cdot \left(\cdots \beta \left(W_{2}^{S}\;\cdot \;\beta \left(W_{1}^{S}\;\cdot \;u_{i}^{B}+b_{1}^{S}\right)+b_{2}^{S}\right)\cdots \right)+b_{L}^{S}$$

where the *W*_*S*_^*S*^ and *b*_*S*_^*S*^ are the weights and biases of the *L* layer neural network, respectively, and *β* is the nonlinear activation function. Equations (1) and (2) represent two multilayer perceptrons with *L* layers, respectively.

The user expression mapped through the base feature space will be used for adversarial learning in the interaction space and adversarial learning in the social space, respectively, which will be introduced below. Therefore, the base feature space and bilateral generative adversarial networks are combined to jointly mine information and improve recommendation performance.

### 3.3. “Interaction Space Adversarial Learning” Module

To better learn user and item representations, we use the generative adversarial network in the interaction space because of its powerful ability to learn complex data distributions to capture users’ preferences in selecting items. As shown in the lower left part of Figure 1, the interaction space adversarial training module consists of two parts: the generator attempts to select as many items that can best match the user’s interests as candidates as possible; the discriminator’s goal is to try to override the candidates generated by the generator. 

#### 3.3.1. The Generator in the Interaction Space

The goal of the generator is to approximate the potential true conditional distribution *P*_*real*_^*I*^ (*v*^*I*^|*u*_*i*_^*I*^) and generate the most relevant candidate samples. First, we use *g*_*score*_^*I*^ (*u*_*i*_^*I*^, *v*_*j*_^*I*^) to denote the item’s *v*_*j*_^*I*^ click or purchase likelihood by the user *u*_*i*_^*I*^, as shown in Equation (3):

(3) $$g_{score}^{I}(u_{i}^{I},\;v_{j}^{I})=\left(u_{i}^{I}\;\cdot \;v_{j}^{I}\right)+\varphi_{g}^{I}$$

where $\varphi_{g}^{I}$ is the bias. After normalizing the probabilities by using the softmax function, we obtain the generator score function in the interaction space $G_{score}^{I}$ as shown in Equation (4):

(4) $$G_{score}^{I}=\frac{exp(g_{score}^{I}(u_{i}^{I},\;\;v_{j}^{I}))}{\sum_{v_{j}\in V}exp(g_{score}^{I}(u_{i}^{I},\;v_{j}^{I}))}$$

Second, we use this score function to obtain the user *u*_*i*_^*I*^ prediction scores for all items *y*_1_^*I*^, *y*_2_^*I*^ ⋯ *y*_*m*_^*I*^ and after sorting these items, we select the items with the top k items as candidate items. 

#### 3.3.2. The Discriminator in the Interaction Space

After the generator generates the candidate items, the discriminator is responsible for overriding the candidate items generated by the generator. The advantage of the popularity sampling method over other common sampling methods lies in its simplicity and ability to handle cold-start problems. So the discriminator improves its discriminative power by utilizing a two-part prevalence-based sampling strategy [28]. The prevalence-based sampling strategy is used to accurately obtain positive and negative example items for adversarial training. The discrimination between positive items, negative items and candidate items is designed for the continuous game between generator *G* and discriminator *D* to better learn the true data distribution in the training data. 

The main process of the popularity-based sampling strategy is as follows. First, the popularity of an item is expressed in terms of the number of users who have interacted with *n*_*j*_. Second, a popularity mean (Mean) is calculated to reflect the average popularity of all items. Items above the mean popularity value are defined as high-popularity items and those below the mean popularity value are defined as low-popularity items. The mean popularity value is calculated in Equation (5).

(5) $$Mean=\frac{1}{J}\sum_{j=1}^{J}n_{j}$$

where the $n_{j}$ is the first *j* the prevalence of the first item, and *J* is the total number of items. 

**Figure 2:**
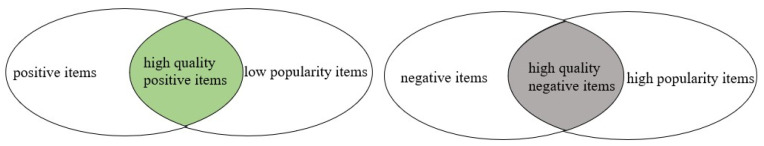
Schematic diagram of prevalence sampling.

According to the definition of popularity, we believe that among the positive examples of items that users have interacted with, the low-popularity items represent the users’ true interest preferences. Similarly, among the negative example items that the user has interacted with, the high popularity items reflect the user’s true aversion tendency. Therefore, the high-quality positive items, $p^{I},$ will be obtained by intersecting the user’s positive items with the low-popularity items, and similarly, the high-quality negative items, $e^{I}$, will be obtained by intersecting the user’s negative example items with the high popularity items, as shown in Figure 2. 

The main idea of discriminating between positive and negative items and candidate items is that users’ preferences for predicted candidate items shall not be higher than the users’ preference for high-quality positive items; the users’ preference for predicted candidate items shall not be lower than the users’ preference for high-quality negative items. 

The score function of the discriminator in the interaction space *D*_*score*_^*I*^ is shown in Equation (6):

(6) $$D_{score}^{I}=\frac{exp(f_{score}^{I}(u_{i},\;v_{j}))}{\sum_{v_{j}\in V}exp(f_{score}^{I}(u_{i},\;v_{j}))}$$

(7) $$f_{score}^{I}(u_{i},v_{j})=\left(u_{i}\;\cdot \;v_{j}\right)+\varphi_{f}^{I}$$

where *φ*_*f*_^*I*^ is the bias. As in Equation (7), we can obtain the prediction score of each item in the discriminator. 

In the stage of training discriminator *D*, the user ratings of high-quality positive and negative example items, as well as candidate items, are fed into the discriminator *D* with the aim of overriding the candidate items generated by the generator. The discriminator loss function $L_{D_{\varphi }}^{I}$ is trained to maximize the difference between users’ ratings of candidate items and users’ ratings of high-quality positive examples and maximize the difference between users’ ratings of candidate items and users’ ratings of high-quality negative examples. The objective function of discriminator *D* is shown in Equation (8):

(8) $$\underset{D_{\varphi }}{\min}L_{D_{\varphi }}^{I}=-E\left[\left(log\sigma \left(y_{p}^{I}-y_{c}^{I}\right)+log\sigma \left(y_{c}^{I}-y_{e}^{I}\right)\right)\right]$$

where the *y*_*p*_^*I*^, *y*_*e*_^*I*^ denote the user’s prediction scores for high-quality positive items and high-quality negative items obtained by using the prevalence-based sampling strategy, and *y*_*c*_^*I*^ denotes the user’s prediction scores for the candidate items generated by the generator. 

In the stage of training the generator *G*, the user’s ratings of high-quality positive example items and candidate items are fed into the generator *G*, with the aim of generating candidate items that better match the user’s true preferences. The difference between the user’s rating of candidate items and the user’s rating of high-quality positive examples is minimized by training, i.e., maximizing the generator loss function $L_{G_{\theta }}^{I}$. The objective function of the generator *G* is shown in Equation (9):

(9) $$\underset{G_{\theta }}{\max}L_{G_{\theta }}^{I}=-E[\log\sigma (y_{p}^{I}-y_{c}^{I})]$$

where *y*_*c*_^*I*^ denotes the user’s the predicted rating of the candidate item, the *y*_*p*_^*I*^ denotes the user’s prediction scores for the positive example items. The generator *G* is trained to fight against the discriminator *D*, until the discriminator *D* cannot distinguish the candidate items from the real data. 

### 3.4. “Social Space Adversarial Learning” Module

In order to better learn user expressions from a social perspective, we utilize another generative adversarial network in social space for social information learning. Again, adversarial learning in the social space contains two parts, a generator and a discriminator, as shown in the lower right part of Figure 1. The generator tries to use the generator score function to select friends that are as similar as possible to the mapped user expressions as candidate friends; the discriminator aims to distinguish candidate friends from real friends by the discriminator score function. 

#### 3.4.1. The Generator in the Social Space

The goal of the generator is to approach the underlying true conditional distribution through adversarial training *P*_*real*_^*S*^ (*f*^*S*^|*u*_*i*_^*S*^) and let the user *u*_*i*_^*S*^ generate the most relevant candidate friends. Similarly, we use *g*_*score*_^*S*^ (*u*_*i*_^*S*^, *f*_*j*_^*S*^) to denote *f*_*j*_^*S*^ is the friend of the user *u*_*i*_^*S*^, as shown in Equation (10):

(10) $$g_{score}^{S}(u_{i},\;k_{j})=\left(u_{i\;}^{S}\cdot \;f_{j}^{S}\right)+\varphi_{g}^{S}$$

where *φ*_*g*_^*S*^ is the bias. After normalizing the probabilities by using the softmax function, we obtain the score function of the generator in the social space *G*_*score*_^*S*^ as shown in Equation (11):

(11) $$G_{score}^{S}=\frac{exp(g_{score}^{S}(u_{i\;}^{S},\;f_{j}^{S}))}{\sum_{k_{j}\in K}exp(g_{score}^{S}(u_{i\;}^{S},f_{j}^{S}))}$$

In the following, we use this score function to arrive at the user $u_{i\;}^{S}$ prediction scores for all friends *y*_1_^*S*^, *y*_2_^*S*^ ⋯ *y*_*n*_^*S*^ and after sorting, we select the top k friends as candidate friends. 

#### 3.4.2. The Discriminator in the Social Space

The goal of the discriminator is to override the candidate friends generated by the generator. The discriminator also consists of two parts: a sampling strategy based on popularity and a method for discriminating between positive and negative examples and candidate friends. 

Similarly, we use a popularity-based sampling strategy to select high-quality positive friends and high-quality negative friends. The high-quality positive friends, $p^{S},$ were obtained by intersecting the user’s friends with the low-popularity friends, and similarly, the high-quality negative friends, $e^{S}$, will be obtained by intersecting the user’s negative friends (friends who have no social relationship with the user) with the high popularity friends.

The main idea of discriminating between high-quality positive and negative example friends, and candidate friends is that the similarity between the user and the predicted candidate friend shall not be higher than the similarity between the user and the high-quality positive example friend, and the similarity between the user and the predicted candidate friend shall not be lower than the similarity between the user and the high quality negative example friend. 

The score function of the discriminator in social space *D*_*score*_^*S*^ is shown in Equation (12):

(12) $$D_{score}^{S}=\frac{exp(f_{score}^{S}(u_{i},\;k_{j}))}{\sum_{k_{j}\in K}exp(f_{score}^{S}(u_{i},\;k_{j}))}$$

(13) $$f_{score}^{S}(u_{i},\;k_{j})=\left(u_{i}\;\cdot \;k_{j}\right)+\varphi_{f}^{S}$$

where *φ*_*f*_^*S*^ is the bias. With Equation (13), we can obtain the predicted scores of the user and each friend in the discriminator. Similarly, the objective function for the social space discriminator *D* adversarial training is shown in Equation (14):

(14) $$\underset{D_{\varphi }}{\min}L_{D_{\varphi }}^{S}=-E\left[\left(log\sigma \left(y_{p}^{S}-y_{c}^{S}\right)+log\sigma \left(y_{c}^{S}-y_{e}^{S}\right)\right)\right]$$

where *y*_*p*_^*S*^, *y*_*e*_^*S*^ denotes the user’s prediction scores for the high-quality positive and high-quality negative friends obtained by using the popularity-based sampling strategy, and *y*_*c*_^*S*^ denotes the user’s prediction scores for the candidate friends generated by the generator. 

In the stage of training the optimized social space generator *G*, the users’ ratings of positive examples and high-quality candidate friends are fed into the objective function of the generator *G*, with the aim of generating candidate friends that better match the users’ true preferences. The objective function for generator *G* is shown in Equation (15):

(15) $$\underset{G_{\theta }}{\max}L_{G_{\theta }}^{S}=-E[\log\sigma (y_{p}^{S}-y_{c}^{S})]$$

where *y*_*c*_^*S*^ denotes the user’s predicted score of the candidate friend, the *y*_*p*_^*S*^ denotes the user’s prediction scores for the positive friend. The generator *G* is trained to fight against the discriminator *D* so that the discriminator *D* cannot distinguish the candidate friends from the real data, and in order to make the candidate friends generated by the generator closer to the real data, then the goal is to make the difference between *y*_*p*_^*S*^ and *y*_*c*_^*S*^ becomes smaller and smaller. Thus, let ℒ_*G*_^*S*^_*θ*_ be maximized. 

### 3.5. Adversarial Training Process of the Model

In order to show the training process of the MBSGAN model more clearly, we present the adversarial training algorithm of the MBSGAN model in Algorithm 1. The training of each cycle is mainly divided into three parts: base feature space mapping, adversarial training in the social space and adversarial training in the interaction space, as shown below.
**Algorithm 1**: MBSGAN adversarial training algorithm.
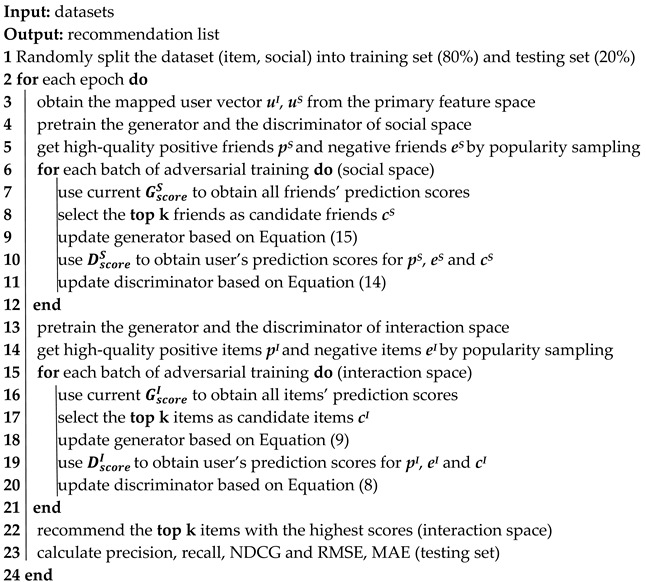


## 4. Experimental Study 

To validate the effectiveness of the MBSGAN model’s performance, the effects of spatial mapping and bilateral adversarial training on model performance are explored, as well as the effects of parameter variations in the model on the results. In this section, two sets of experiments are analyzed in Section 4.2 and Section 4.3 to verify the effectiveness of MBSGAN model performance by analyzing the social recommendation model and the adversarial training recommendation model; model ablation experiments are compared in Section 4.4 to verify the effects of vector mapping and bilateral adversarial training on the model; finally, the selection of the number of candidate samples k values is analyzed in Section 4.5 to verify the effects of model parameter variations on MBSGAN model performance. 

### 4.1. Dataset and Evaluation Metrics 

In this work, four benchmark datasets, Douban, FilmTrust, Ciao, and Epinions, are used to study the performance of the proposed MBSGAN. The Douban data comes from Douban, which contains users’ ratings of movies and social information among users; FilmTrust is a movie dataset from the FilmTrust website, which also contains users’ ratings of movies and social information among users; Ciao comes from an online social platform, which includes users’ ratings of purchased products and social information among users; the Epinions dataset comes from an online social platform where people can review products, which includes users’ ratings of products and social information among users; The specific statistics of the four public datasets are shown in Table 1. 

To evaluate the performance of the model, the evaluation metrics are Precision@ k, Recall@ k, Normalized Cumulative Discount Gain@ k, Mean Absolute Error MAE (Mean Absolute Error), and Root Mean Squared Error RMSE (Root Mean Squared Error). In the top k recommendation task, k is taken as 10 to calculate the first three metrics, and the evaluation metrics are shown below. 

Precision: the proportion of all predicted positive samples that contain true positive samples. The definition is as follows:

(16) $$precision=\frac{TP}{TP+TN}$$

where *TP* (True Positive) represents the number of positive samples predicted as positive and *FP* (False Positive) represents the number of negative samples predicted as positive. 

*Recall* (recall): the proportion of true positive samples that are predicted to be positive, which is defined as follows.

(17) $$Recall=\frac{TP}{TP+FN}$$

where *FN* (False Negative) represents the number of negative samples predicted as negative. Recall@ k represents the proportion of true positive samples that are predicted as positive in the first k samples. 

Normalized discounted cumulative gain (*NDCG*) is a composite assessment score that evaluates the combined quality of relevance and ranking of items in the test set in the top k recommendation list. Higher NDCG values indicate better ranking results.

(18) $$NDCG=\frac{DGG}{IDGG}$$

(19) $$DCG=\sum_{i=1}^{\left|REL\right|}\frac{2^{{rel}_{i}}-1}{\log_{2}\left(i+1\right)}$$

where |*REL*| denotes the results are sorted in the order of relevance from largest to smallest in the best way. *rel*_*i*_ denotes the relevance score of item *i*. *DCG* (discounted cumulative gain) calculates the score of items in user *u*’s recommendation list by considering both relevance and order factors, and *IDCG* (ideal discounted cumulative gain) is the result of *DCG* normalization. 

Mean absolute error (*MAE*): the mean value of the error between the model predicted scores and the true scores, reflecting the degree of similarity between the predicted scores and the true scores. The definition is as follows:

(20) $$MAE=\frac{\sum_{\left(u,i\right)\in R_{test}}\left|r_{ui}-r_{ui}^{\prime }\right|}{\left|R_{test}\right|}$$

where |*R*_*test*_| denotes the number of user ratings of items in the test set, the $r_{ui}$ and $r_{ui}^{\prime }$ are the real ratings and the ratings predicted by the algorithm, respectively. 

Root mean squared error (*RMSE*) is the square root of the ratio of the square of the predicted score to the true score error to the number of observations *n*, as defined below:

(21) $$RMSE=\sqrt{\frac{\sum_{(u,i)\in R_{test}}{\left(r_{ui}-r_{ui}^{\prime }\right)}^{2}}{\left|R_{test}\right|}}$$

When *precision*, *recall*, and *NDCG* values are larger, it indicates better recommendation performance. *MAE* and *RMSE* reflect the difference between predicted and true scores, and smaller values indicate higher accuracy of recommendations. 

### 4.2. Parameter Settings

The parameter settings in the experiment are shown in Table 2. ***k*** is the number of candidate samples, ***d*** denotes the vector dimension, ***λ*** is the regularization coefficient, ***batch*** is the batch size, and ***lr*** is the learning rate. In the experiment, the number of epochs for Douban and FilmTrust was set to 30, and ciao was set to 40.

### 4.3. Experimental Comparison of Social Recommendation Models 

To demonstrate the advantages of the MBSGAN model proposed in this paper over other social recommendation models, the experimental results of the MBSGAN model are compared with eight baseline social recommendation models on four publicly available datasets. Among them, SBPR and SoMA are Bayesian-based social recommendation models; Diffnet++, Light_NGSR, and GNN-DSR are graph convolutional neural network-based social recommendation models; RSGAN, DASO, and ESRF are social recommendation models incorporating generative adversarial networks. Each of the eight baseline social recommendation models is described as follows:

(1) SBPR [15] (2014): for the first time, social relationships were added to the Bayesian personalized ranking algorithm (BPR), arguing that users are more biased towards items preferred by their friends than items with negative feedback or no feedback.
(2) SoMA [29] (2022): a social recommendation model based on the Bayesian generative model that exploits the displayed social relationships and implicit social structures among users to mine their interests.
(3) DiffNet++ (2020): a social recommendation model using graph convolutional networks, by aggregating higher-order neighbors in the social relationship graph and item interaction graph, respectively, and by distinguishing the influence of neighbors on users with an attention mechanism.
(4) Light_NGSR [30] (2022): a social recommendation model based on the GNN framework, which retains only the neighborhood aggregation component and drops the feature transformation and nonlinear activation components. It aggregates higher-order neighborhood information from user–item interaction graphs and social network graphs.
(5) GNN-DSR [31] (2022): a social recommendation model using graph convolutional networks, which considers dynamic and static representations of users and items and combines their relational influences. It models the short-term dynamic and long-term static interaction representations of user interest and item attractiveness, respectively.
(6) RSGAN (2019): a social recommendation model that uses GAN and social reconstruction, where generators generate items that friends interact with as items that users like, and discriminators are used to distinguish items that friends interact with from items that users really like themselves.
(7) DASO (2019): a social recommendation model based on GAN that fuses heterogeneous information by mapping each other in interaction space and social space. The generator picks samples that are likely to be of interest to users, and the discriminator distinguishes between real samples and generated samples.
(8) ESRF (2020): a social recommendation model using generative adversarial networks and social reconstruction, where the generator generates friends with similar preferences to the user and the discriminator distinguishes between the user’s personal preferences and the average preferences of friends.

To verify the effectiveness of MBSGAN combined with vector mapping and bilateral generative adversarial networks, we separate the experimental results into two different types according to the two main tasks of the recommender system: “Top-N recommendation” and “rating prediction”. Meanwhile, since the SoMA, Light_NGSR, and GNN-DSR codes are not available, we only compare the MAE and RMSE metrics on the Ciao and Epinions datasets, as shown in Table 3. 

The MBSGAN model was compared with five social information-based recommendation models with the following results: 

By observing the experimental results in Table 3, it can be seen that the MBSGAN proposed in this paper obtains optimal values in terms of each metric in the Douban, FilmTrust, and Ciao datasets compared to the baseline model. Further analysis of the experimental results leads to the following conclusions: Diffnet++, RSGAN, DASO, ESRF, and MBSGAN perform better compared to the traditional social recommendation method SBPR because the four baseline models of the latter incorporate the network model in deep learning, because deep learning models have multiple layers and nonlinear activation functions that can capture complex nonlinear relationships between users and projects. Traditional recommendation models often rely on linear or shallow models, which cannot effectively capture the complex and nonlinear nature of user–item interactions. And compared with SBPR, which only considers the first-order neighbors of users, the use of network models can tap more information about user–item interactions and the association information in social relationships to obtain a richer user representation. Compared with RSGAN and ESRF using GAN, DASO and MBSGAN outperform these two models in all metrics, indicating that RSGAN and ESRF share the same user representation in both interaction and social spaces, which limits the learning of user representation, while DASO and MBSGAN learn user representation in the social space and interaction space separately to learn more fully the information in each space. This is because learning user expressions separately can reduce irrelevant interference. Separating user representations in social spaces and interaction spaces can avoid interference between spaces and improve the independence and accuracy of the model for information in each space. The MBSGAN model performs better than DASO, demonstrating the effectiveness of basic feature space mapping.

By observing the experimental results in Table 4, we can see that, compared with the baseline model, the MBSGAN proposed in this paper obtains the better result in terms of MAE metrics of the Ciao dataset and on the MAE and RMSE metrics of Epinions. Further analysis of the experimental results leads to the following conclusions: compared with SoMA, Light_NGSR, and GNN-DSR, which use only social relationships, the experimental results of MBSGAN on two real datasets almost outperform these baseline models, indicating that the application of generative adversarial networks in social recommendation is beneficial to improving the accuracy of the models and reducing scoring errors. 

### 4.4. Experimental Comparison of Pairwise Training Recommendation Models 

To demonstrate the advantages of the MBSGAN model proposed in this paper over other generative adversarial network-based recommendation models, the experimental results of the MBSGAN model are compared with six baseline adversarial training recommendation models on three publicly available datasets. Among them, CFGAN, GCGAN, and GANRec [27] are collaborative filtering recommendation models based on generative adversarial networks, and RSGAN, DASO, and ESRF are social recommendation models based on generative adversarial networks. The other three baseline adversarial training recommendation models that are different from the social recommendation model experiments are described as follows:

(1) CFGAN (2018): a collaborative filtering recommendation model based on generative adversarial networks, where the generator generates the user’s purchase vector, and the discriminator is responsible for distinguishing between the generator’s “fake” purchase vector and the real user’s purchase vector.
(2) GCGAN (2021): Based on CFGAN, the discriminator captures the latent features of users and items through a graph convolutional network to distinguish whether the input is a “fake” purchase vector by the generator or a real user purchase vector.
(3) GANRec (2023): a collaborative filtering model based on generative adversarial networks, where the generator picks out items that the user may like as negative samples and the discriminator distinguishes between real positive samples and generator-generated negative samples.

In order to verify the effectiveness of MBSGAN combined with vector mapping and bilateral generative adversarial networks, we divided the experimental results into two different types according to the two major tasks of the recommendation systems: “Top-N recommendation” and “rating prediction”, respectively. The results of comparing the MBSGAN model with several generative adversarial network-based recommendation models on the Top-N recommendation task were as follows.

By observing the experimental results in Table 5 and Table 6, it is evident that the proposed MBSGAN obtains optimal values for each metric in the Douban, FilmTrust, and Ciao datasets compared to the six baseline models. Further analysis of the experimental results leads to the following conclusions: compared with the three collaborative filtering recommendation models CFGAN, GCGAN, and GANRec, RSGAN, DASO, and ESRF perform better because the latter three models incorporate social information, indicating that the proper use of social relationships can help alleviate the sparsity problem and lead to more accurate recommendation results. A social relationship is a direct relationship between people. The addition of social relationships provides more information and basis for recommendation algorithms, making the recommendation results more accurate. Compared with RSGAN and ESRF, DASO and MBSGAN outperformed them on almost all three datasets, indicating that constructing bilateral generative adversarial networks in both spaces can more fully exploit the information in the interaction and social spaces than unilateral adversaries, thus improving the accuracy of the models and reducing scoring errors. This is because the bilateral adversarial network not only mines the interaction information in the interaction space, but also uses it to learn information in the social space, alleviating the noise problem in both spaces and improving recommendation accuracy.

### 4.5. Comparison of Ablation Experiments of Models 

**Figure 3:**
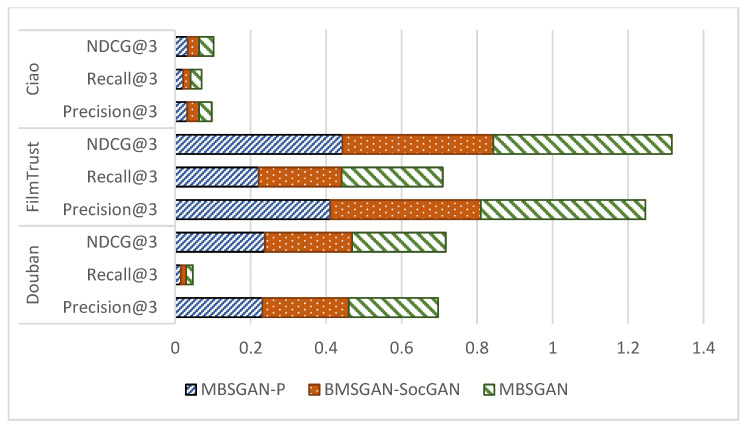
Comparison of ablation experimental results of MBSGAN model (Top-N recommendation).

**Figure 4:**
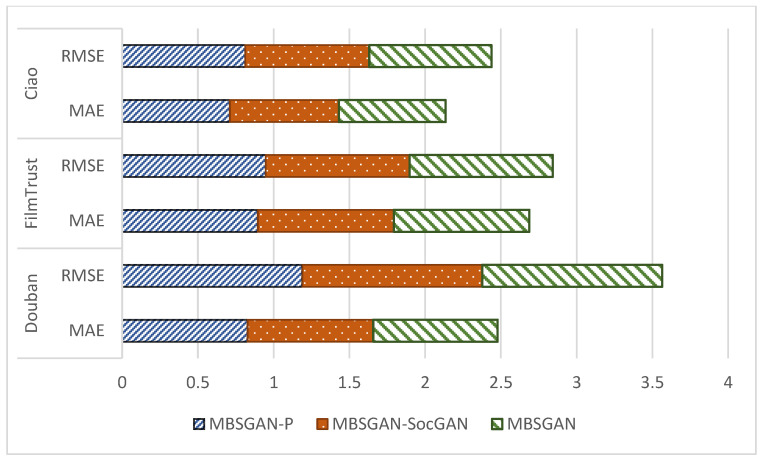
Comparison of ablation experimental results of MBSGAN model (score prediction).

In order to verify the effectiveness of introducing spatial mapping and bilateral generative adversarial networks in the model, this paper compares the MBSGAN model with the MBSGAN-P model with the vector mapping being removed, and with the MBSGAN-SocGAN model with the social spatial adversarial learning being removed, through ablation experiments. The comparison results are shown in Figure 3 and Figure 4, respectively. 

By analyzing the experimental results presented in Figure 3 as well as Figure 4, it can be observed that, after removing the spatial vector mapping part of the base features or bilateral generative adversarial networks, the experimental results of each metric become worse on all three datasets, indicating that both of the above modules have a positive impact on the model performance. The introduction of the spatial mapping part better explores the common features behind different user interactions, which leads to more accurate user expressions. The basic feature space mapping can help the model better discover and extract the common features of users in different spaces. By integrating and mapping user characteristics across different spaces, it is possible to model the similarities and correlations between users in different spaces, thereby more accurately capturing user interests and preferences. In addition, it can be seen that the model performance decreases if the bilateral generative adversarial networks are not used, indicating that using generative adversarial networks to learn users’ social information is helpful to obtaining more accurate user expressions. The discriminator network in GAN can evaluate the difference between the generated social information and the real social information. By continuously optimizing the adversarial process between the generator and the discriminator, the generated social information can be made closer to the real social information, thereby improving the accuracy and credibility of user expression.

### 4.6. Effect of the Number of Candidate Samples k Values 

**Figure 5:**
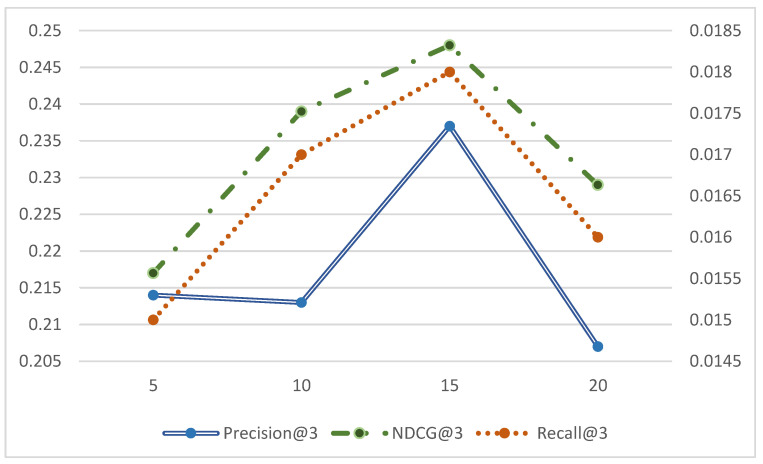
Experimental performance of MBSGAN models with different k values (Douban).

**Figure 6:**
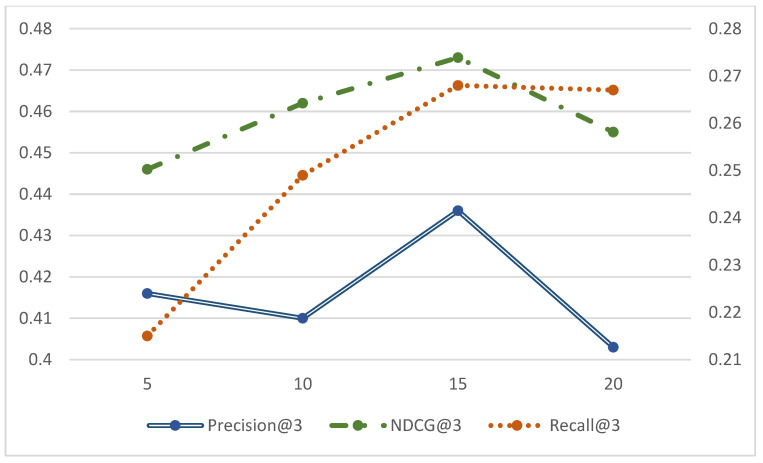
Experimental performance of MBSGAN model with different k values (FilmTrust).

**Figure 7:**
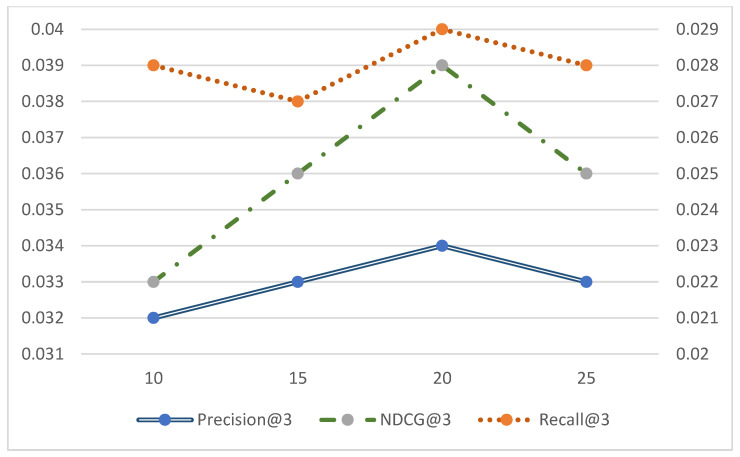
Experimental performance of MBSGAN model with different k values (Ciao).

The k value is the number of candidate samples in interaction-space adversarial learning as well as social-space adversarial learning, and is used to discriminate among the three in the discriminator of the two spaces together with the high-quality positive and negative examples obtained from sampling, thus enabling the generator to more accurately select candidate samples for recommendation. In order to investigate the effect of the number of candidate samples k value on the model performance, different k values are selected to examine the performance of the proposed MBSGAN model in this paper on three publicly available datasets, and then a reasonable k value is selected as the number of candidate samples to be selected. The experimental results of the MBSGAN model corresponding to different k values are shown in Figure 5, Figure 6 and Figure 7. 

In order to present the results of Precision@3, Recall@3, and NDCG@3 with the number of candidate samples clearly in the same plot, the horizontal coordinates are set as k values and the vertical coordinates are the evaluation values, here the vertical coordinates are used as the primary and secondary axes. The blue line represents Precision@3, the green line represents NDCG@3, and the orange line represents Recall@3. In Figure 5, Figure 6 and Figure 7, the values of Precision@3 and NDCG @3 are based on the main axis on the left, and the Recall@3 values are based on the secondary axis on the right. 

Analyzing Figure 5, Figure 6 and Figure 7, it can be observed that the experimental results of the MBSGAN model are affected by the number of candidates k, which shows different trends on the three datasets. The model works best when k = 15 on the Douban dataset, when k = 15 on the FilmTrust dataset, and when k = 20 on the Ciao dataset. When the value of k chosen is too small, fewer candidate samples, positive and negative examples are utilized and the interaction information cannot be more fully utilized. And when the k value chosen is too large, it leads to overfitting and makes the recommendation results inaccurate. 

### 4.7. Convergence of the Model

To verify the convergence of the model, we conducted experiments on three datasets: Douban, Ciao, and FilmTrust to obtain the learning curve of the MBSGAN model. Among them, the principal axis represents precision@3 and NDCG@3. The secondary coordinate axis represents recall@3 and the horizontal axis represents the number of epochs.

**Figure 8:**
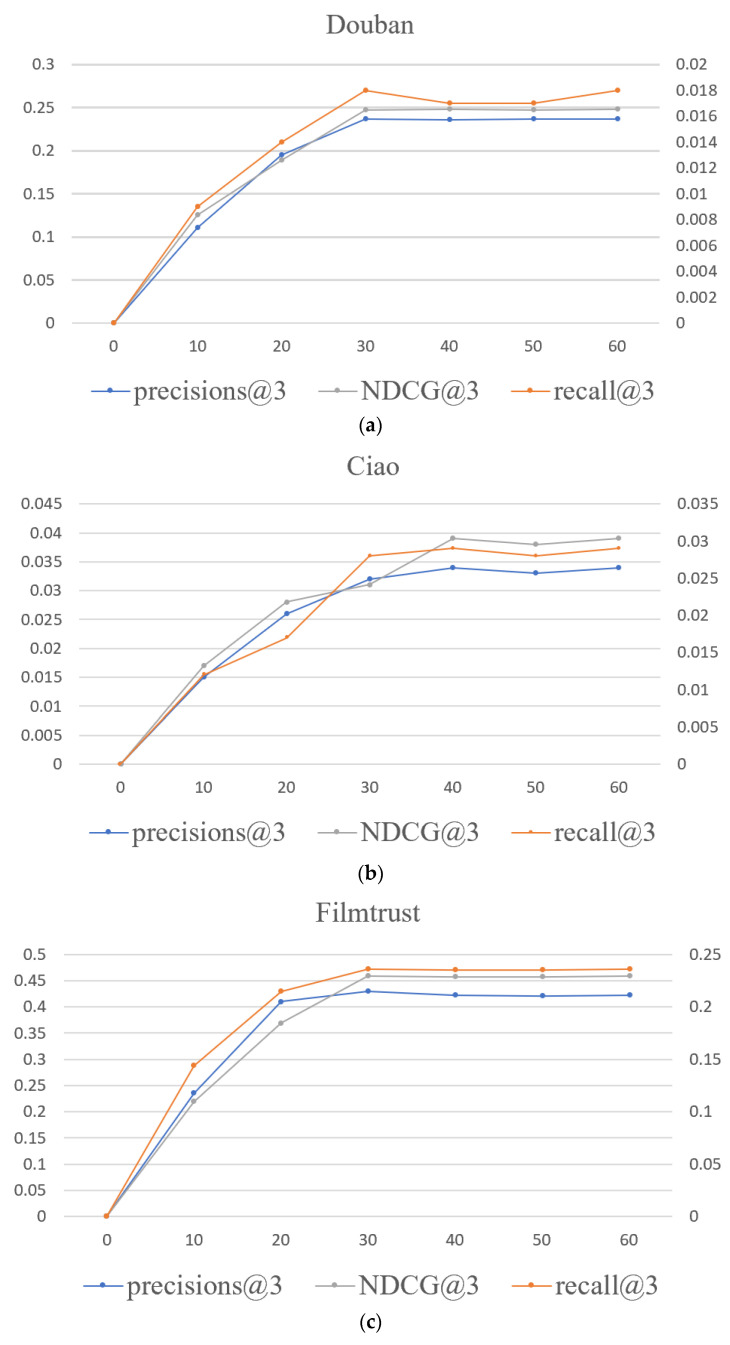
The learning curve of MBSGAN on three datasets. (**a**) convergence of the model on the Douban dataset. (**b**) convergence of the model on the Ciao dataset. (**c**) convergence of the model on FilmTrust dataset.

From Figure 8, it can be seen that the MBSGAN model has achieved convergence on all three datasets. Among them, on the Douban and FilmTrust datasets, the model converges when the number of epochs reaches 30, and on the Ciao dataset, the model converges when the number of epochs reaches around 40.

## 5. Conclusions

In this paper, we propose a recommendation model based on spatial mapping and bilateral generative adversarial networks (MBSGAN). We first map the base feature space to the interaction space and social space, respectively, to achieve the fusion of heterogeneous spaces and obtain more accurate user representations in both spaces. Then, bilateral generative adversarial networks are constructed in the interaction space and social space to learn the complex information in the respective spaces. Through two sets of comparative experiments, the effectiveness of using the base feature space to fuse heterogeneous information was demonstrated, and the advantages of our constructed bilateral generative adversarial networks in mining information were also verified. However, the factors that affect user interaction behavior are diverse and complex. We only consider the impact of user social information on recommendations, which is not comprehensive enough to learn the potential interaction characteristics of users. We should also consider more diverse information, such as item attribute information and user’s own attribute information. Therefore, in the next work, we should consider fusing more auxiliary information for user expression and item expression in bilateral generative adversarial networks, such as knowledge graph information or user attribute information. At the same time, it is necessary to find appropriate fusion methods for this information to further enrich the feature representation of users and items, thereby improving the accuracy of recommendations.

## Figures and Tables

**Table 1:** Dataset statistics.

Data Items	User Volume	Item Volume	Rating Amount	Social Relationships
Douban	2848	39,586	894,887	35,770
FilmTrust	1508	2071	35,497	1853
Ciao	7375	105,114	284,086	111,781
Epinions	40,163	139,738	664,824	442,980

**Table 2:** Parameter Settings.

Dataset	*k*	*D*	*λ*	*batch*	*Lr*
Douban	15	32	1 × 10^−7^	512	5 × 10^−5^
FilmTrust	15	32	1 × 10^−6^	512	5 × 10^−5^
Ciao	20	32	2 × 10^−5^	1024	5 × 10^−4^
Epinions	20	32	2 × 10^−5^	1024	5 × 10^−4^

**Table 3:** Experimental results of social recommendation model (Top-N recommendation).

Model	Douban			FilmTrust			Ciao		
	Precision@3	Recall@3	NDCG@3	Precision@3	Recall@3	NDCG@3	Precision@3	Recall@3	NDCG@3
SBPR	0.182	0.013	0.208	0.221	0.094	0.267	0.022	0.008	0.024
DiffNet++	0.204	0.016	0.220	0.375	0.201	0.416	0.025	0.012	0.028
RSGAN	0.211	0.015	0.217	0.347	0.203	0.385	0.029	0.014	0.033
DASO	0.224	0.017	0.239	0.400	0.234	0.445	0.033	0.023	0.038
ESRF	0.223	0.017	0.238	0.380	0.232	0.392	0.032	0.016	0.037
MBSGAN	0.237	0.018	0.248	0.430	0.236	0.459	0.034	0.029	0.039

**Table 4:** Experimental results of social recommendation model (rating prediction).

Model	Ciao MAE	RMSE	MAE	Epinions RMSE
SoMA	0.785	0.998	1.050	1.189
Light_NGSR	0.736	0.973	0.835	1.084
GNN-DSR	0.697	0.944	0.801	1.057
MBSGAN	0.704	0.807	0.765	0.931

**Table 5:** Experimental results of the recommendation model based on adversarial training (Top-N recommendation).

Model	Douban			FilmTrust			Ciao		
	Precision@3	Recall@3	NDCG@3	Precision@3	Recall@3	NDCG@3	Precision@3	Recall@3	NDCG@3
CFGAN	0.203	0.011	0.204	0.239	0.073	0.252	0.023	0.011	0.025
RSGAN	0.211	0.015	0.217	0.347	0.203	0.385	0.029	0.014	0.033
DASO	0.224	0.017	0.239	0.380	0.234	0.392	0.033	0.023	0.037
ESRF	0.223	0.017	0.238	0.400	0.232	0.445	0.032	0.016	0.038
GCGAN	0.190	0.014	0.218	0.212	0.229	0.229	0.021	0.010	0.022
GANRec	0.204	0.015	0.217	0.249	0.231	0.230	0.022	0.011	0.026
MBSGAN	0.237	0.018	0.248	0.436	0.268	0.473	0.034	0.029	0.039

**Table 6:** Experimental results of the recommendation model based on adversarial training (score prediction).

Model	Douban		FilmTrust		Ciao	
	MAE	RMSE	MAE	RMSE	MAE	RMSE
CFGAN	1.233	1.529	0.981	1.151	1.199	1.423
RSGAN	1.255	1.561	1.022	1.370	1.245	1.560
DASO	0.883	1.224	0.994	1.101	0.859	1.228
ESRF	0.900	1.256	1.683	1.849	1.701	1.869
GCGAN	0.898	1.253	0.956	1.005	0.889	1.255
GANRec	0.922	1.215	1.001	1.059	0.998	1.253
MBSGAN	0.820	1.187	0.895	0.946	0.704	0.807
